# Supplementary material for: Design of Quercetin-Loaded Natural Oil-Based Nanostructured Lipid Carriers for the Treatment of Bacterial Skin Infections
Source: Molecules. 2022 Dec 12;27(24):8818. doi: 10.3390/molecules27248818 (PMC9785768; doi:10.3390/molecules27248818)
Supplement: Supplementary file 1 [file molecules-27-08818-s001.zip › molecules-2068219-supplementary.pdf]

S1 – Supplementary Material

Influence of QR concentration on average particle size

A case of study QR-SF-NLC

1)

			Size (d.nm):	% Intensity:	St Dev (d.nm):
Z-Average (d.nm):	155,6	Peak 1:	205,0	100,0	87,76
PdI:	0,225	Peak 2:	0,000	0,0	0,000
Intercept:	0,852	Peak 3:	0,000	0,0	0,000
Result quality :	Good				

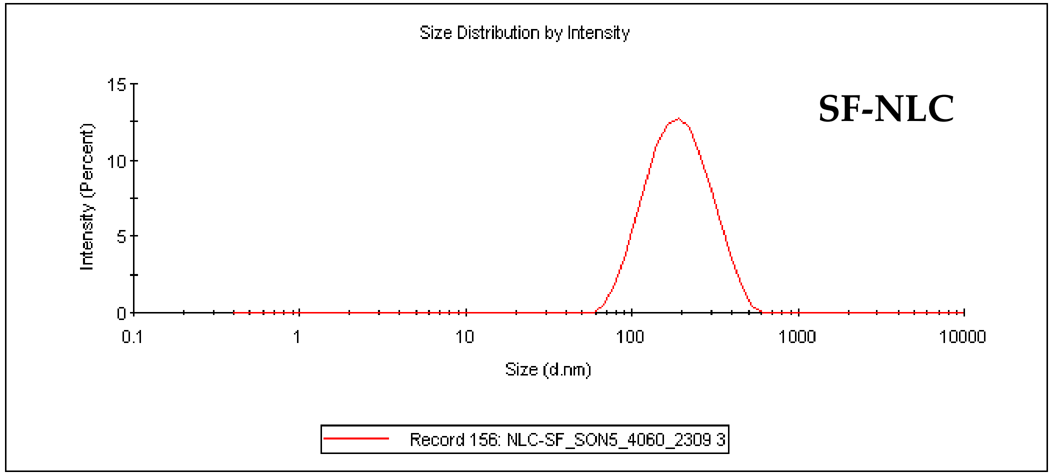

2)

			Size (d.nm):	% Intensity:	St Dev (d.nm):
Z-Average (d.nm):	142,6	Peak 1:	230,7	89,8	125,9
PdI:	0,411	Peak 2:	31,24	8,7	8,282
Intercept:	0,728	Peak 3:	4758	1,5	746,0
Result quality :	Good				

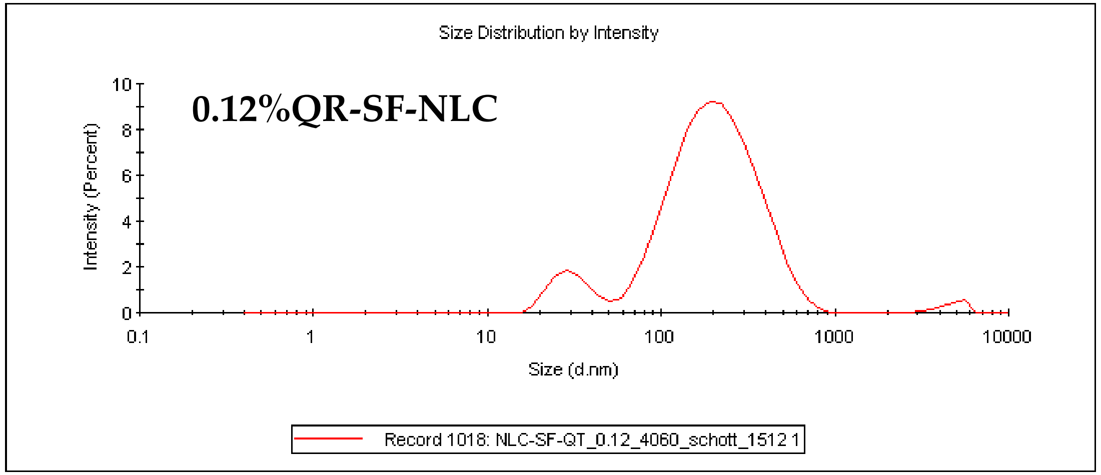

3)

			Size (d.nm):	% Intensity:	St Dev (d.nm):
Z-Average (d.nm):	260,8	Peak 1:	472,4	83,2	370,0
PdI:	0,451	Peak 2:	48,95	9,6	14,24
Intercept:	0,690	Peak 3:	4056	7,2	1052
Result quality :	Good				

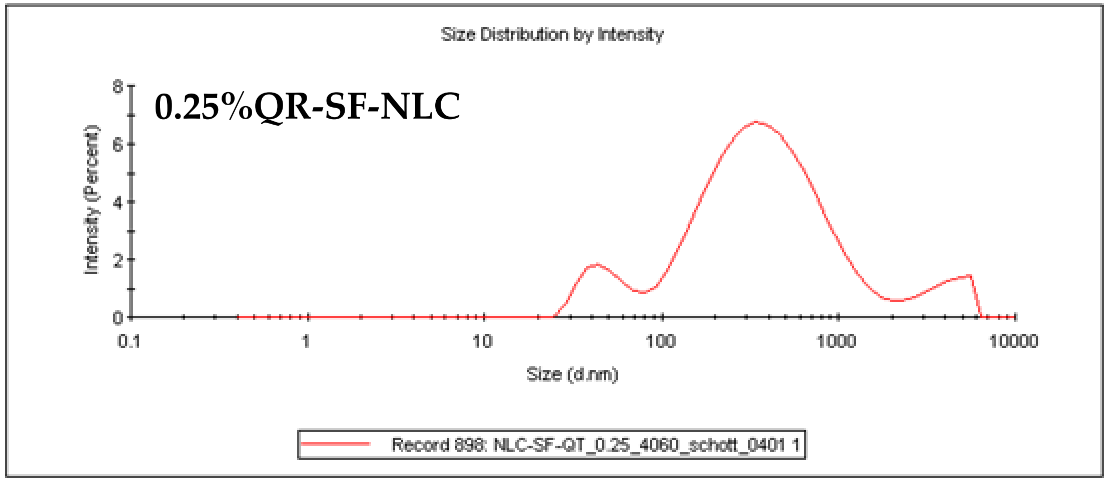

4)

			Size (d.nm):	% Intensity:	St Dev (d.nm):
Z-Average (d.nm):	156,2	Peak 1:	284,7	97,8	232,0
PdI:	0,466	Peak 2:	4362	2,2	929,6
Intercept:	0,691	Peak 3:	0,000	0,0	0,000
Result quality :	Good				

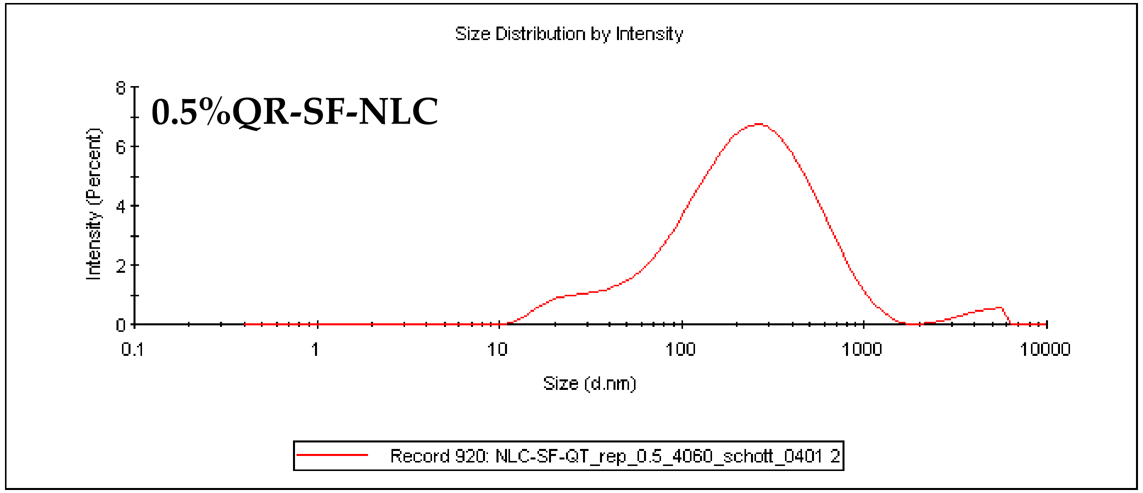

			Size (d.nm):	% Intensity:	St Dev (d.nm):
Z-Average (d.nm):	204,0	Peak 1:	446,0	77,6	278,7
PdI:	0,497	Peak 2:	68,72	18,7	22,97
Intercept:	0,685	Peak 3:	4688	2,4	791,6
Result quality :	Good				

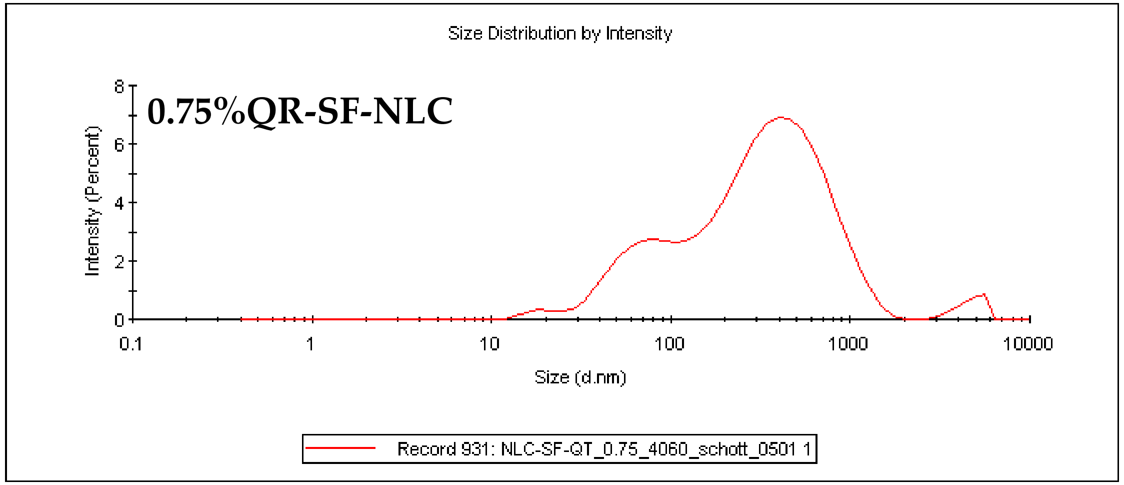

			Size (d.nm):	% Intensity:	St Dev (d.nm):
Z-Average (d.nm):	193,5	Peak 1:	616,7	91,1	819,8
PdI:	0,550	Peak 2:	37,96	8,9	11,34
Intercept:	0,684	Peak 3:	0,000	0,0	0,000
Result quality :	Good				

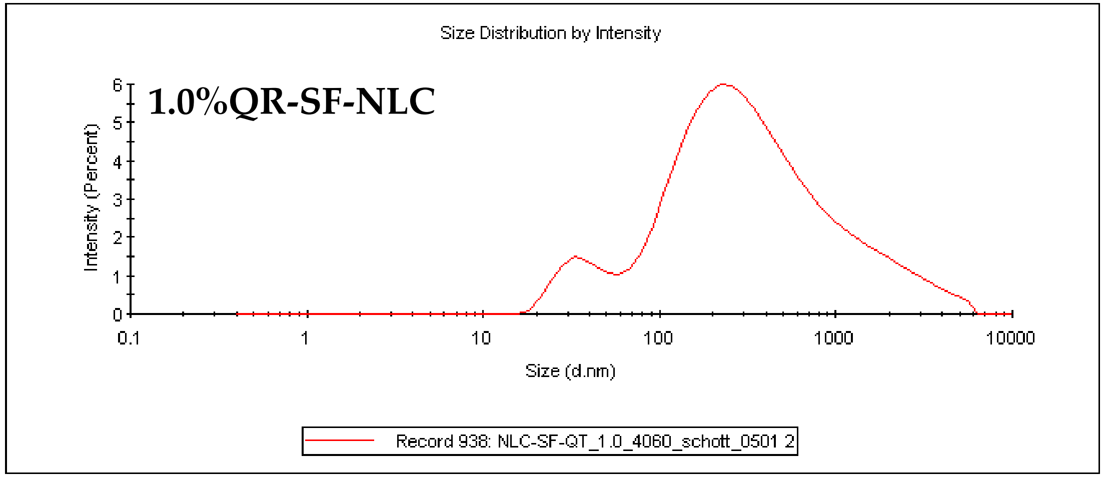

			Size (d.nm):	% Intensity:	St Dev (d.nm):
Z-Average (d.nm):	232,1	Peak 1:	755,6	91,7	1007
PdI:	0,497	Peak 2:	42,21	8,3	10,56
Intercept:	0,683	Peak 3:	0,000	0,0	0,000
Result quality :	Good				

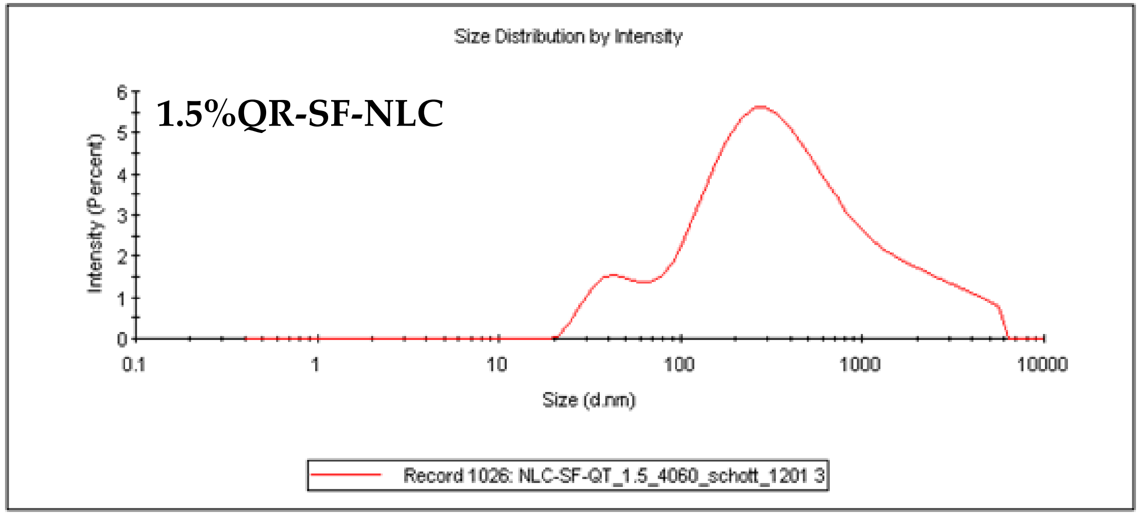

			Size (d.nm):	% Intensity:	St Dev (d.nm):
Z-Average (d.nm):	288,7	Peak 1:	235,1	49,0	110,9
PdI:	0,689	Peak 2:	1437	32,8	724,5
Intercept:	0,685	Peak 3:	4362	12,0	875,2
Result quality :	Good				

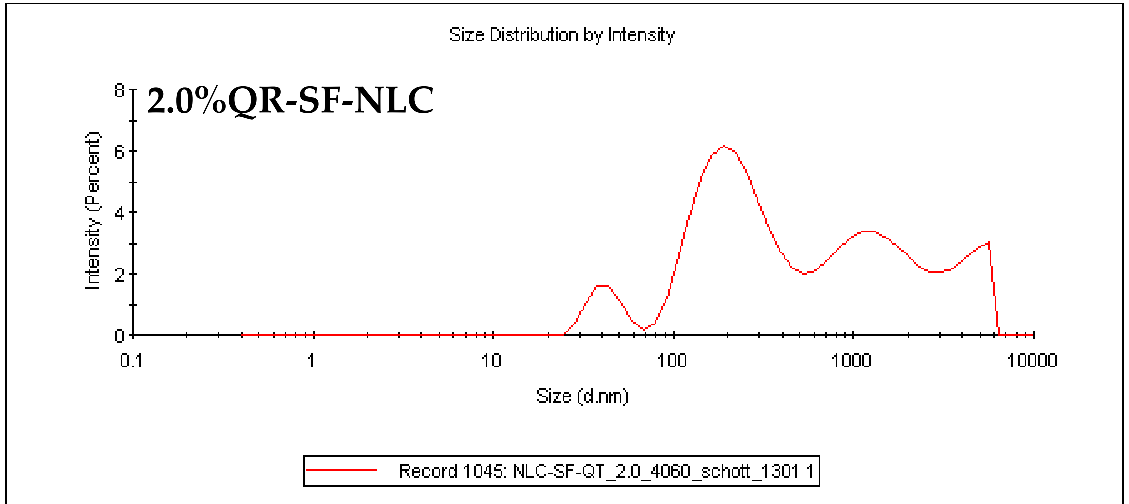

			Size (d.nm):	% Intensity:	St Dev (d.nm):
Z-Average (d.nm):	231,7	Peak 1:	550,0	83,0	554,5
PdI:	0,731	Peak 2:	4168	9,6	989,3
Intercept:	0,681	Peak 3:	37,19	7,4	7,683

Result quality : Refer to quality report

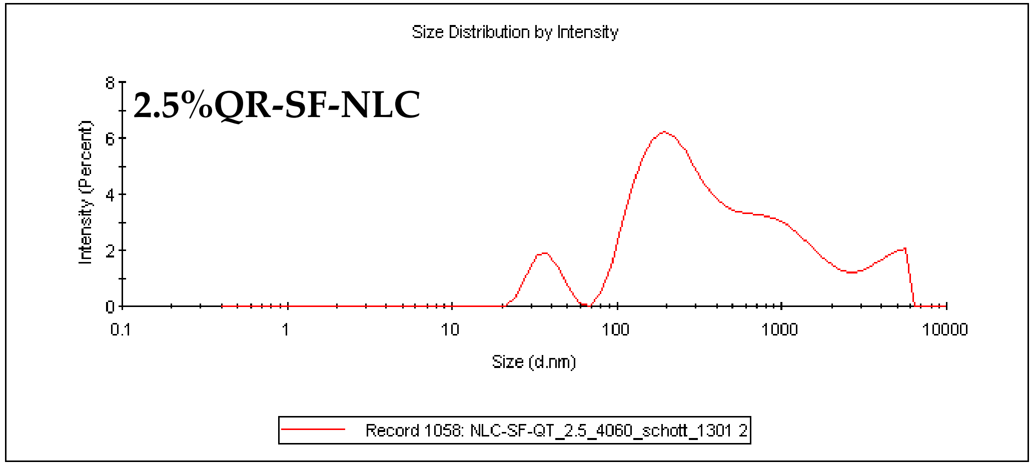

			Size (d.nm):	% Intensity:	St Dev (d.nm):
Z-Average (d.nm):	236,7	Peak 1:	424,8	86,7	385,8
PdI:	0,747	Peak 2:	4049	13,3	1119
Intercept:	0,677	Peak 3:	0,000	0,0	0,000

Result quality : Refer to quality report

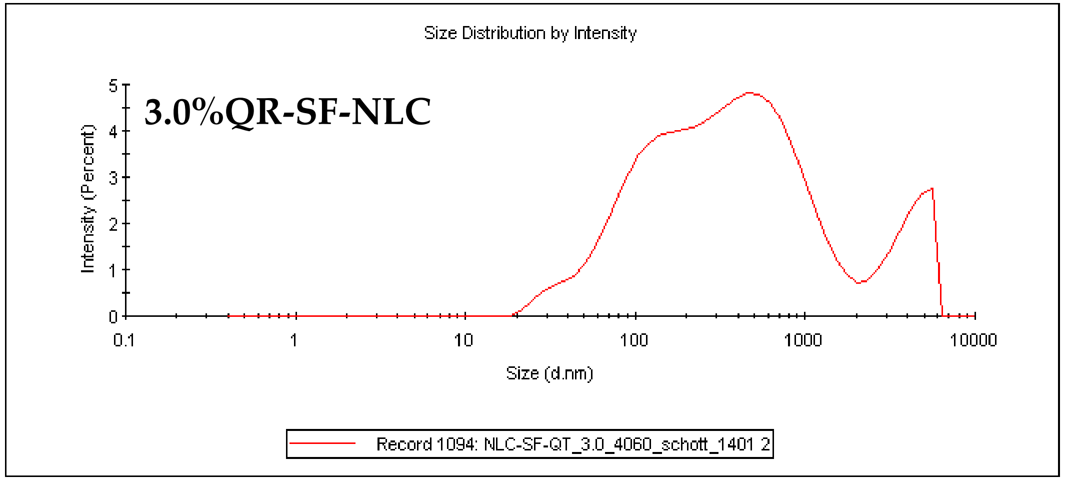

			Size (d.nm):	% Intensity:	St Dev (d.nm):
Z-Average (d.nm):	395,0	Peak 1:	605,9	53,5	241,7
PdI:	0,615	Peak 2:	136,0	35,9	63,50
Intercept:	0,676	Peak 3:	5196	10,6	477,5

Result quality : Refer to quality report

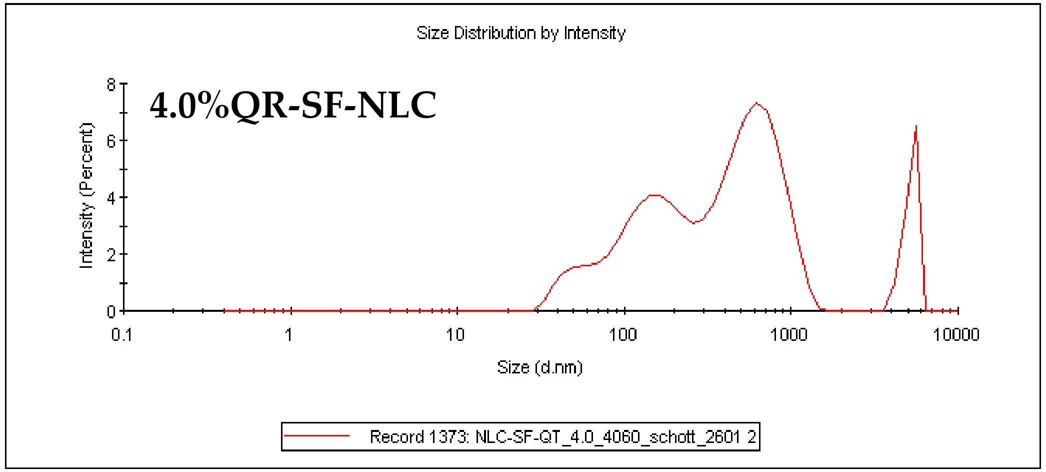

			Size (d.nm):	% Intensity:	St Dev (d.nm):
Z-Average (d.nm):	488,1	Peak 1:	598,9	51,9	220,7
PdI:	0,649	Peak 2:	161,9	44,4	74,37
Intercept:	0,664	Peak 3:	5560	3,7	6,104e-5

Result quality : Refer to quality report

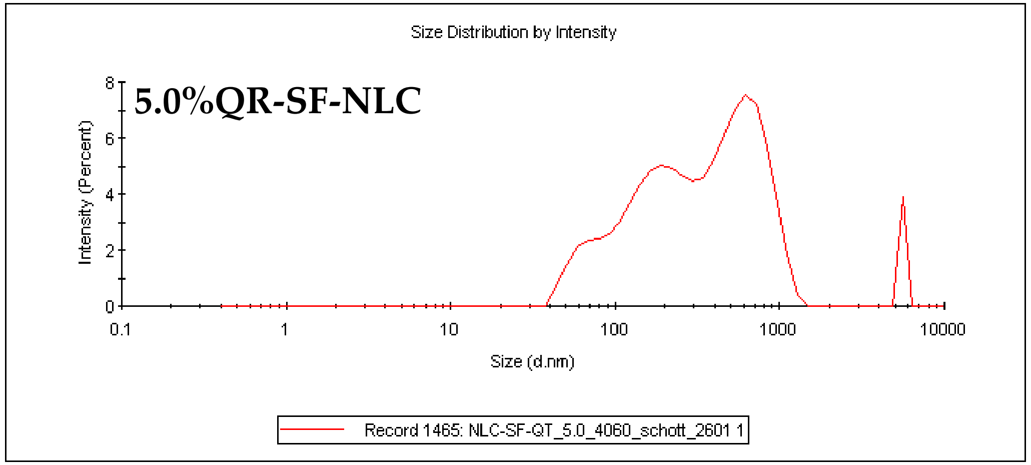

S1 – Supplementary Material

Influence of QR concentration on Z-potential

A case of study QR-SF-NLC

			Mean (mV)	Area (%)	St Dev (mV)
Zeta Potential (mV):	-46,2	Peak 1:	-46,2	100,0	7,58
Zeta Deviation (mV):	7,58	Peak 2:	0,00	0,0	0,00
Conductivity (mS/cm):	0,0109	Peak 3:	0,00	0,0	0,00

Result quality : Good

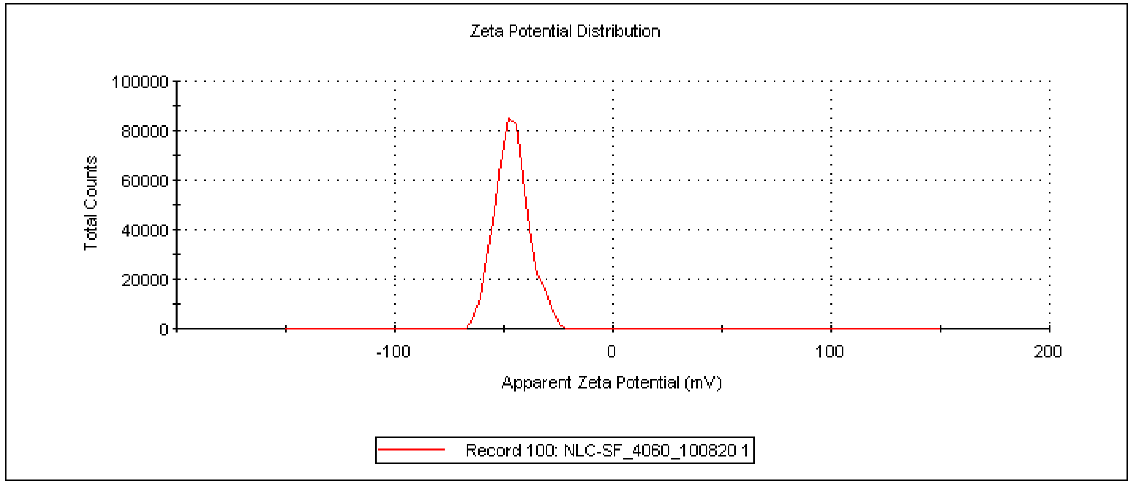

			Mean (mV)	Area (%)	St Dev (mV)
Zeta Potential (mV):	-45,7	Peak 1:	-47,3	93,7	10,3
Zeta Deviation (mV):	12,9	Peak 2:	-13,0	5,8	7,00
Conductivity (mS/cm):	0,0102	Peak 3:	-83,5	0,5	2,61

Result quality : See result quality report

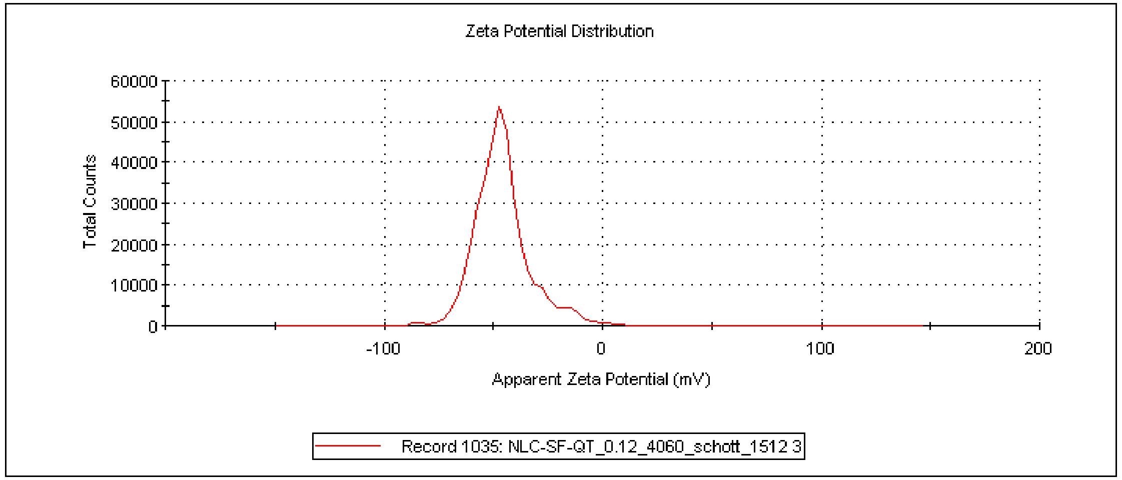

			Mean (mV)	Area (%)	St Dev (mV)
Zeta Potential (mV):	-45,7	Peak 1:	-47,3	93,7	10,3
Zeta Deviation (mV):	12,9	Peak 2:	-13,0	5,8	7,00
Conductivity (mS/cm):	0,0102	Peak 3:	-83,5	0,5	2,61

Result quality : See result quality report

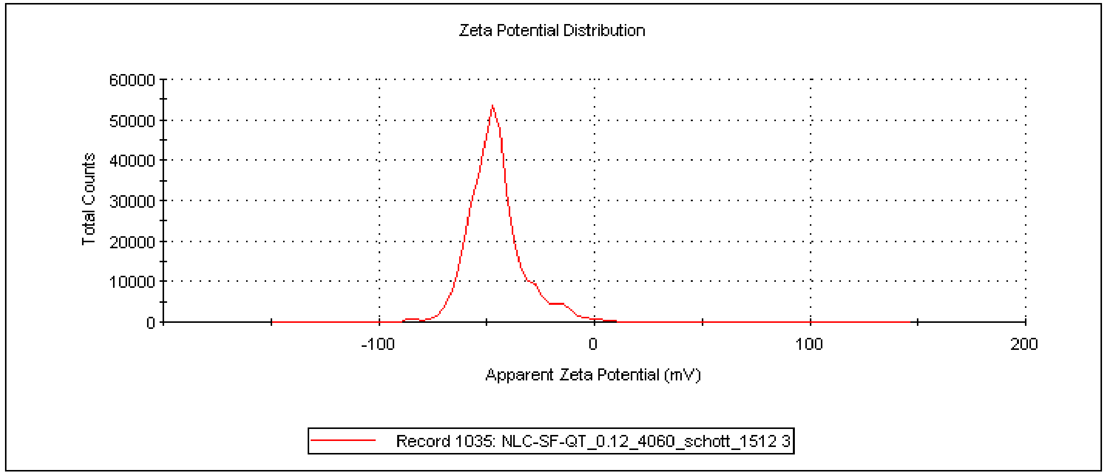

			Mean (mV)	Area (%)	St Dev (mV)
Zeta Potential (mV):	-46,4	Peak 1:	-34,6	89,5	10,5
Zeta Deviation (mV):	36,9	Peak 2:	-130	3,4	4,96
Conductivity (mS/cm):	0,0232	Peak 3:	0,0859	3,0	4,46

Result quality : See result quality report

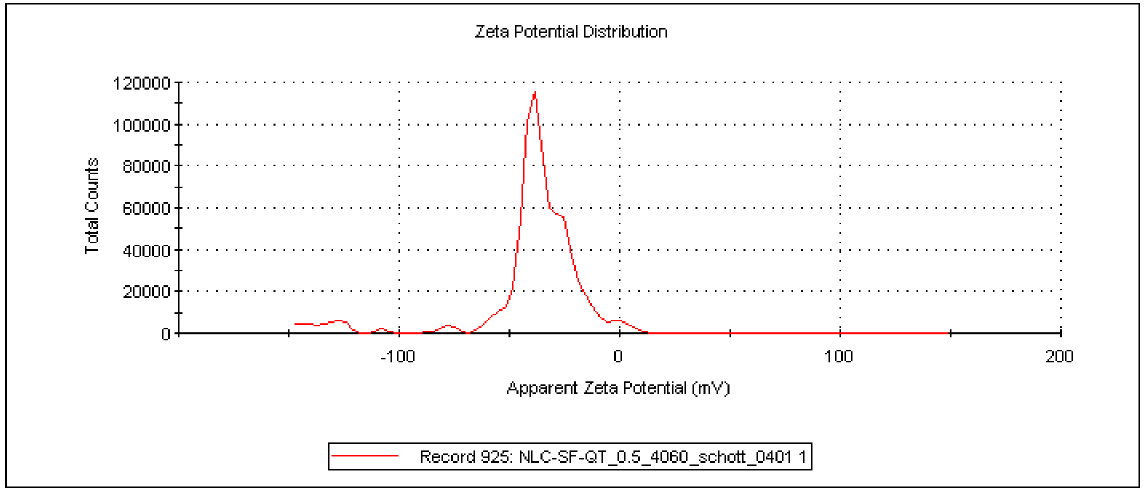

			Mean (mV)	Area (%)	St Dev (mV)
Zeta Potential (mV):	-51,0	Peak 1:	-24,7	65,4	9,07
Zeta Deviation (mV):	49,8	Peak 2:	-45,3	12,0	3,64
Conductivity (mS/cm):	0,00506	Peak 3:	-70,6	11,9	8,48

Result quality : See result quality report

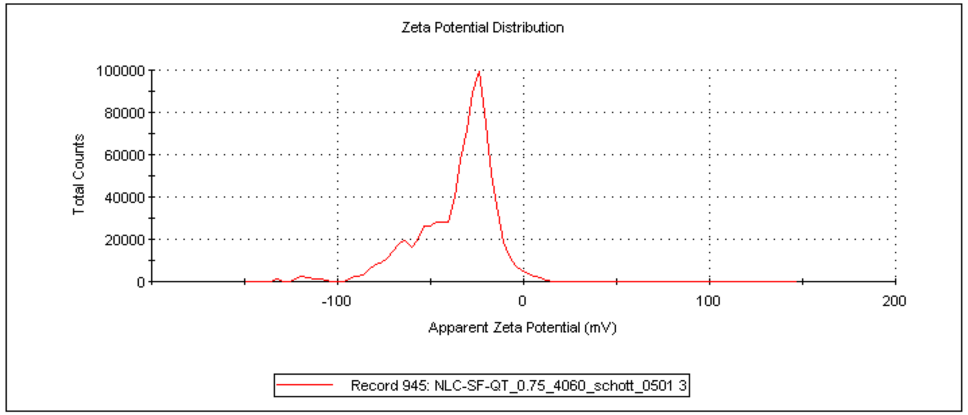

			Mean (mV)	Area (%)	St Dev (mV)
Zeta Potential (mV):	-48,1	Peak 1:	-42,0	100,0	10,7
Zeta Deviation (mV):	30,5	Peak 2:	0,00	0,0	0,00
Conductivity (mS/cm):	0,00500	Peak 3:	0,00	0,0	0,00

Result quality : See result quality report

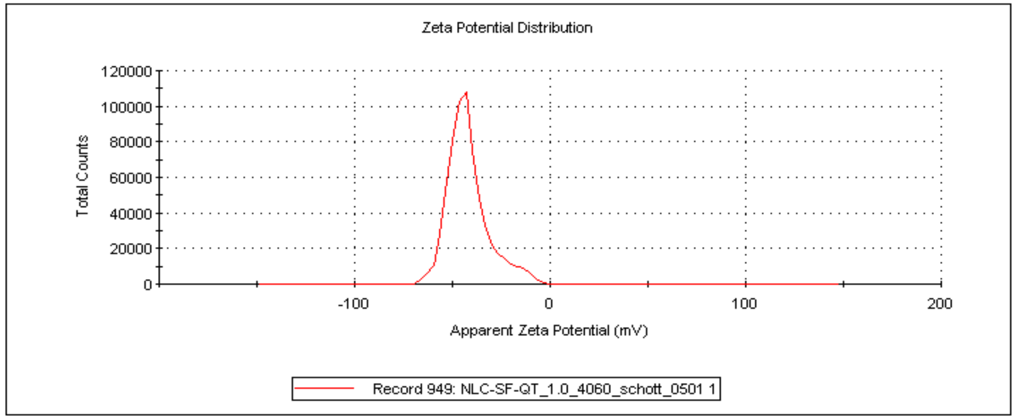

			Mean (mV)	Area (%)	St Dev (mV)
Zeta Potential (mV):	-47,1	Peak 1:	-34,9	100,0	6,04
Zeta Deviation (mV):	42,2	Peak 2:	0,00	0,0	0,00
Conductivity (mS/cm):	0,00801	Peak 3:	0,00	0,0	0,00

Result quality : See result quality report

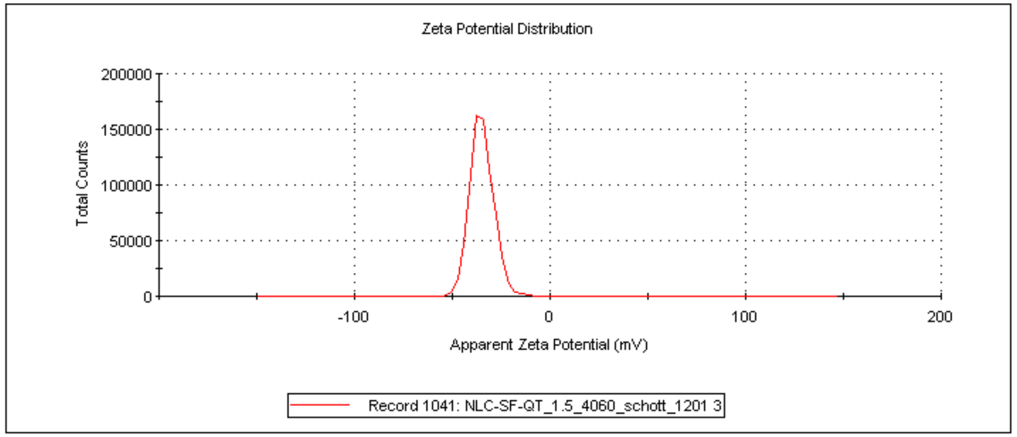

			Mean (mV)	Area (%)	St Dev (mV)
Zeta Potential (mV):	-47,5	Peak 1:	-33,0	86,4	10,5
Zeta Deviation (mV):	38,7	Peak 2:	-113	11,0	7,70
Conductivity (mS/cm):	0,0151	Peak 3:	1,90	2,5	4,09

Result quality : See result quality report

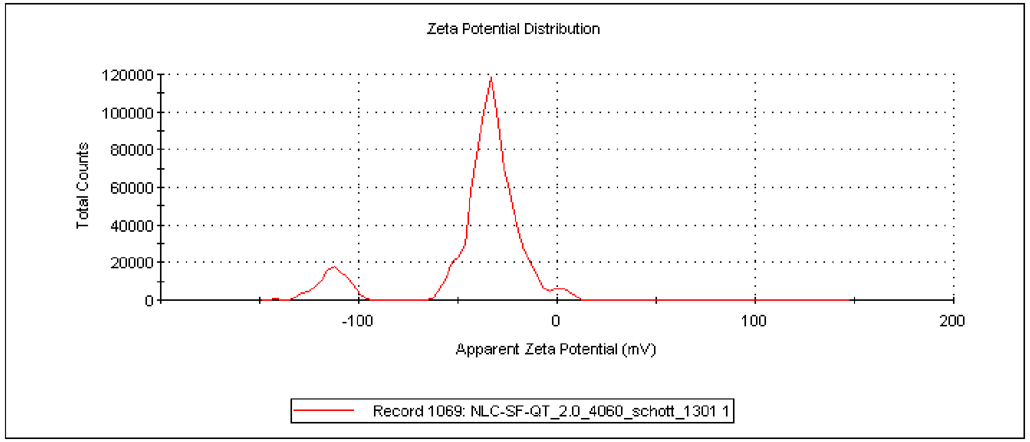

			Mean (mV)	Area (%)	St Dev (mV)
Zeta Potential (mV):	-42,4	Peak 1:	-43,4	97,3	11,0
Zeta Deviation (mV):	12,5	Peak 2:	-6,15	1,5	3,36
Conductivity (mS/cm):	0,0148	Peak 3:	3,84	1,2	3,57

Result quality : See result quality report

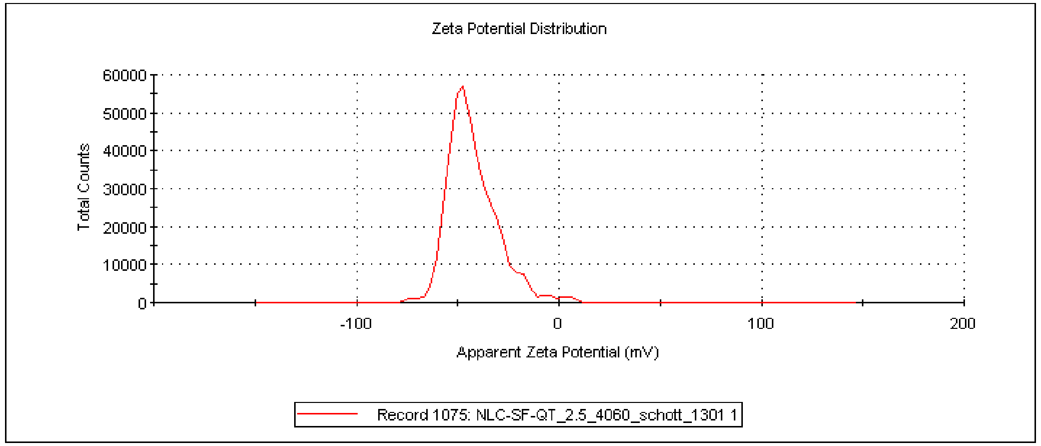

			Mean (mV)	Area (%)	St Dev (mV)
Zeta Potential (mV):	-51,0	Peak 1:	-51,0	100,0	5,45
Zeta Deviation (mV):	5,45	Peak 2:	0,00	0,0	0,00
Conductivity (mS/cm):	0,0103	Peak 3:	0,00	0,0	0,00

Result quality : Good

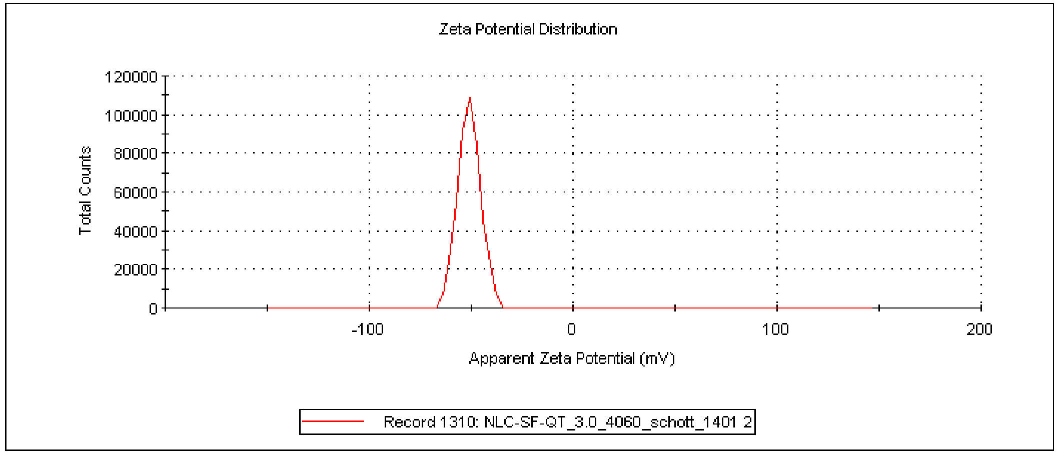

			Mean (mV)	Area (%)	St Dev (mV)
Zeta Potential (mV):	-42,4	Peak 1:	-33,3	100,0	9,11
Zeta Deviation (mV):	43,4	Peak 2:	0,00	0,0	0,00
Conductivity (mS/cm):	0,0125	Peak 3:	0,00	0,0	0,00

Result quality : See result quality report

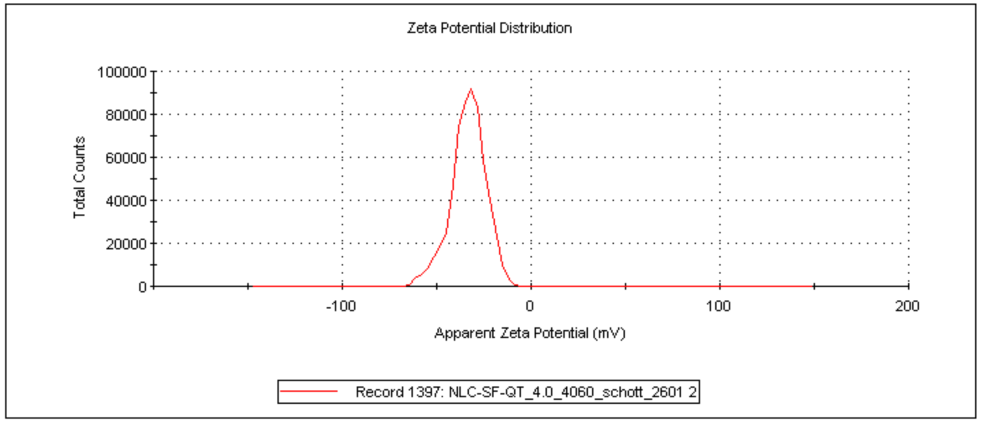

			Mean (mV)	Area (%)	St Dev (mV)
Zeta Potential (mV):	-52,0	Peak 1:	-9,69	100,0	6,56
Zeta Deviation (mV):	81,8	Peak 2:	0,00	0,0	0,00
Conductivity (mS/cm):	0,0145	Peak 3:	0,00	0,0	0,00

Result quality : Good

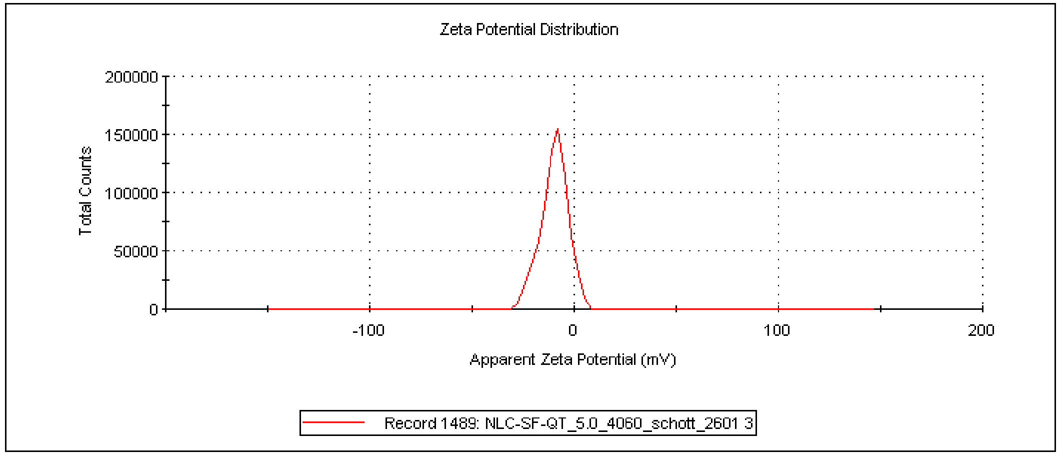

S2 – Supplementary Material

- Table S2 - Solubility of QR in NLCs excipients

Excipient	QR, µ/mg
**Sunflower oil**	5
**Olive oil**	3
**Corn oil**	5
**Coconut oil**	63
**Castor oil**	1633
**Myristic acid**	60
**Water with S80**	15
**Water**	2
